# Allergic Conjunctivitis at Sheikh Zayed Regional Eye Care Center, Gambia

**Published:** 2012-01

**Authors:** Patricia D Wade, Anthonia N Iwuora, Laritza Lopez, Mustapha A Muhammad

**Affiliations:** Sheikh Zayed Regional Eye Care Center, Gambia

**Keywords:** Prevalence, Allergy, Conjunctivitis

## Abstract

**Purpose:**

To assess the prevalence of allergic conjunctivitis in Gambia and to determine its epidemiologic features, seasonal variations and associated ocular and systemic conditions.

**Methods:**

Records of patients clinically diagnosed with allergic conjunctivitis between April 2007 and March 2008 were reviewed. Variables including age, sex, date of presentation, and systemic and ocular findings were recorded.

**Results:**

A total of 7,912 patients were visited within the study period, out of which 624 (7.9%) were diagnosed with allergic conjunctivitis. The disease was equally distributed in male and female subjects, with high preponderance in children (54.5%). Most patients (60.7%) presented during the dry season as compared to the rainy season. Refractive error was the most common ocular condition associated with the condition present in 7.4% of patients while the most common systemic association was asthma, reported in 1.4% of cases.

**Conclusion:**

Allergic conjunctivitis in Gambia is more common in children than in adults and has seasonal variation with more patients presenting during dry seasons. Refractive errors are the most common ocular problem associated with the disease and asthma is a systemic association.

## INTRODUCTION

Allergic conjunctivitis refers to a group of disorders affecting the ocular surface usually associated with type 1 hypersensitivity reactions.^1^,^2^ It comprises a spectrum of disorders from non-sight threatening conditions such as seasonal allergic conjunctivitis (SAC), perennial allergic conjunctivitis (PAC), giant papillary conjunctivitis (GPC), to less common but sight threatening diseases such as vernal keratoconjunctivitis (VKC), and atopic keratoconjunctivitis (AKC).^3^ Along with an IgE mediated reaction, complex chronic inflammation is involved in the pathogenesis of many ocular allergies.^4^ Allergic conjunctivitis is a typically mast cell mediated hypersensitivity reaction. Exposure of sensitized, IgE coated mast cells to airborne allergens is the primary irritating stimulus. Chain reactions in the plasma membrane of mast cells lead to membrane rupture and extrusion of the contents of the cell into surrounding tissues. These contents act as mediators and together with many other cell mediators stimulate the proliferation of fibroblasts and recruitment of various cell types to the conjunctiva, all of which result in development of papillae as seen in patients with vernal keratoconjunctivitis.^5^ Topical antihistamines have been employed for many years to provide fast relief for patients. These include mast cell stabilizers such as sodium cromoglicate, nedocromil and lodoxamide which also have anti-inflammatory effects.^6^ Allergic conjunctivitis has a global distribution with a widely varying incidence. It is less common in Northern Europe and North America, but more common in Africa, Mediterranean countries, Central and South America and in the Indian subcontinent.^7^

## METHODS

This retrospective study was conducted at Sheikh Zayed Regional Eye Care Center and was approved by the ethical committee of this institution. This center is the only tertiary eye care facility in Gambia overseeing the activities of seven secondary centers from where patients are referred. Patients may also come directly to the center without referral.

Records of patients visited from April 2007 to March 2008 and diagnosed with allergic conjunctivitis were retrieved for the purpose of the study. Follow-up cases were not included in the study. Patient information including age, sex and address were recorded. Other data included presenting complaints and their duration, drug and family history, and previous allergies or history of asthma. Findings on slit lamp and fundus examination were noted. Patients with tarsal papillae, pigmented conjunctiva, limbal papillae associated with follicles, conjunctival scarring, trichiasis or corneal scarring were diagnosed with allergic conjunctivitis concomitant with trachoma.

Obtained information was recorded into data sheets and entered into Epi-info software version 3.0. Frequency values were tested using the Chi-square test. Significance level was set at 0.05.

## RESULTS

**Figure 1.**
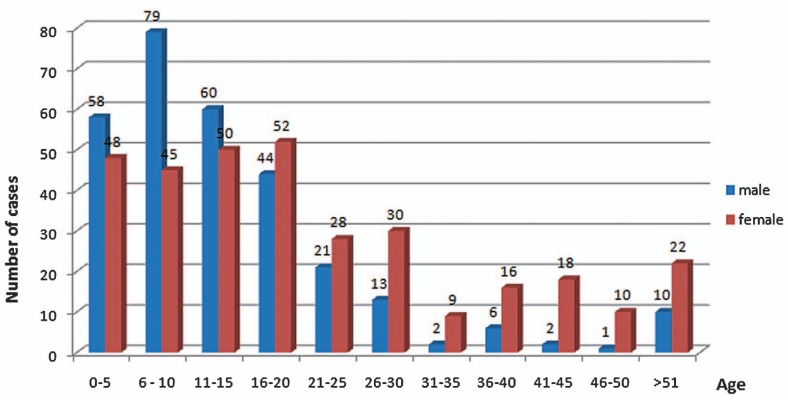
Age-sex distribution of cases

A total of 1,248 eyes of 624 patients were diagnosed with allergic conjunctivitis within the study period. Figure 1 shows the age and gender distribution; 340 patients (54.5%) were children aged 0–15 years. The child to adult ratio was 1:1.2 (P=0.002). There were 296 (47.4%) male and 328 (52%) female subjects with 1:1.1 female/male ratio reflecting no statistically significant difference by gender (P=0.07).

**Figure 2.**
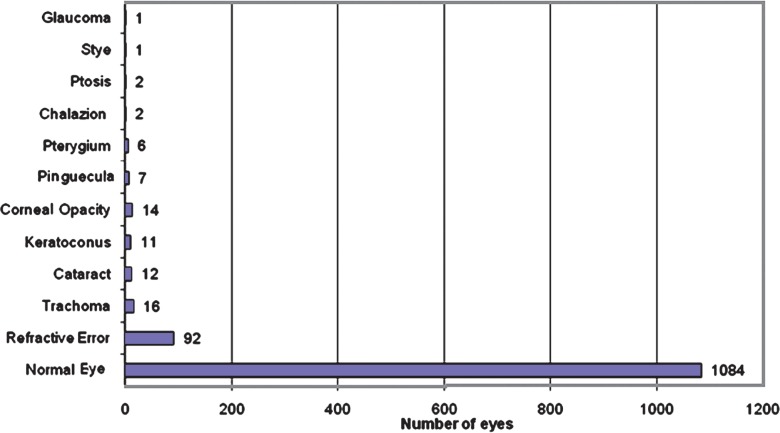
Concomitant ocular conditions

Figure 2 demonstrates the ocular associations of allergic conjunctivitis. Refractive errors were present in 92 (7.4%) eyes while trachoma was diagnosed in 16 (1.3%) eyes. Corneal opacities were present in 14 (1.1%) eyes and cataracts were observed in 12 (1.0%) eyes; 11 eyes (0.9%) had keratoconus while 7 eyes (0.6%) had pinguecula. Pterygia were noted in 6 (0.5%) eyes while chalazia and ptosis were present in 2 eyes (0.2%) each. Stye and glaucoma were diagnosed in one eye (0.1%) each.

**Figure 3.**
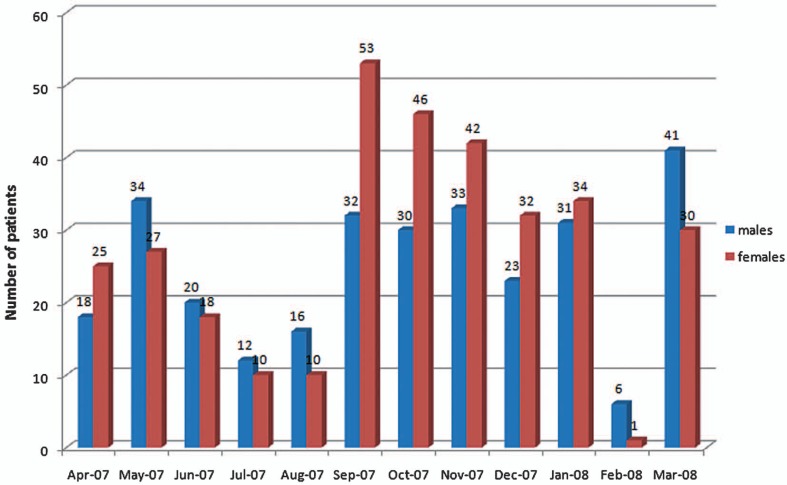
Monthly distribution of cases

Figure 3, shows the monthly distribution of cases; 379 patients (60.7%) were visited during the dry season, November to May, while 245 patients (39.3%) were seen during the rainy season, June to October, providing a ratio of 1:1.5 (P=0.01).

Table 1, shows patients with concomitant systemic diseases. Some patients had other systemic disorders that may or may not be associated with allergic conjunctivitis. Nine patients (1.4%) were asthmatic, while atopic dermatitis, epilepsy, hypertension and diabetes mellitus were diagnosed in one patient (0.2%) each.

## DISCUSSION

Allergic conjunctivitis is one of the most common ocular conditions encountered by pediatricians, internists, and family practitioners.^8^–^10^ Of the 7,912 patients visited within the study period, 624 (7.9%) were diagnosed with various forms of ocular allergies making it one of the most common disorders at our clinic. Although more female subjects were seen with allergy, the gender difference failed to reach statistical significance. This is in line with a similar study performed by Uchio et al who reported female predominance^11^ in ocular allergy but in contrast to the study by Marback et al in which male preponderance was observed.^12^

Gambia has a sandy environment. Children playing outdoors in the sand use their dusty hands to wipe their eyes, introducing foreign substances which affect the conjunctiva, resulting in allergic conjunctivitis.^13^ Allergic conjunctivitis has been noted as one of the most frequent reasons for a child visiting an ophthalmologist.^14^

Gambia has a subtropical climate with distinct cold and hot seasons. From November to May the weather is cold, dry and dusty while the rainy season is from June to October. The majority of patients (60.7%) with allergic conjunctivitis were seen during the dry season, while 39.3% were seen during the rainy season; a finding which can be attributed to the presence of dust and pollen in the air during the dry seasons.

The diagnosis of allergic conjunctivitis at this center was similar to other eye centers in African countries, i.e. by meticulous questioning, emphasizing on the existence of ocular itching and looking for tarsal papillae, follicles and conjunctival pigmentation.^16^ We lacked facilities for specific IgE and skin tests as recommended in other studies.^9^,^13^ There were other ocular conditions coexisting with allergic conjunctivitis; refractive errors were found to be the most common. Mimura et al^17^ in their study on the relationship between refractive errors and allergic conjunctivitis noted a significant number of allergic conjunctivitis patients with refractive errors (22.1%) and concluded that refractive error may be a risk factor for allergic conjunctivitis. Another coexisting condition was trachoma. Based on the 1987 national survey in Gambia, trachoma was one of the leading causes of treatable and avoidable blindness in the country, accounting for 17% of cases. With adequate intervention, the disease has now been reduced to less than 5% in endemic communities. Few cases of trachoma are sometimes seen concomitant with allergic conjunctivitis as in the present study. This confirms the study of Bezerra et al^18^ in Brazil in which in about 5.8% of cases, trachoma coexisted with allergic conjunctivitis.

The use of traditional medicines is quite common in our community as in most other African settings. Harmful eye medications are known to cause irreversible damage to the cornea; 1.1% of our cases were diagnosed with corneal opacities. It is unknown if these opacities were due to traditional eye remedies or because patients with vernal conjunctivitis may also develop central corneal changes known as vernal plaques. These plaques consist of mucin and epithelial cells and can cause scarring and visual loss.^8^ Videokeratography is a useful technique in detecting mild keratoconus, which is a complication of vernal keratoconjunctivitis. In the absence of videokeratography, patients in our study were diagnosed clinically; thereby mild cases were missed. This accounts for a low prevalence (0.9%) of the condition in our series as compared to the study by Totan et al^19^ in which a complementary device was used for diagnosis and reported a prevalence of 26.8% for keratoconus.

Allergic conjunctivitis is a frequent disorder and often associated with other manifestations of atopy such as rhinitis or asthma.^20^ In a study on children with allergic conjunctivitis at an outpatient clinic in Denmark, 24% were found to have asthma concomitant with allergic conjunctivitis while 30% had eczema and 42% had rhinitis.^21^ In our study 1.4% of cases had asthma while only 0.2% had atopic dermatitis. No patient was diagnosed with rhinitis which seems to be a more common association with allergic conjunctivitis. It can be concluded that there is a need to ask patients or look for associated conditions for quick intervention so patients can be managed with other specialties. This may reduce frequent clinic visits and avoid seeking alternative treatment.

## Figures and Tables

**Table 1.** Patients diagnosed with systemic diseases

**Systemic diseases**	**Number**	**Percent**
Asthma	9	1.4
Atopic dermatitis	1	0.2
Epilepsy	1	0.2
Hypertension	1	0.2
Diabetes	1	0.2
